# Interlaced X-ray diffraction computed tomography

**DOI:** 10.1107/S160057671600131X

**Published:** 2016-03-01

**Authors:** Antonios Vamvakeros, Simon D. M. Jacques, Marco Di Michiel, Pierre Senecal, Vesna Middelkoop, Robert J. Cernik, Andrew M. Beale

**Affiliations:** a Department of Chemistry, University College London, 20 Gordon Street, London WC1H 0AJ, England; b Research Complex at Harwell, Rutherford Appleton Laboratory, Didcot, Harwell, Oxfordshire OX11 0FA, England; c School of Materials, University of Manchester, Manchester M13 9PL, England; d ESRF – The European Synchrotron, Grenoble F-38000, France; e Flemish Institute for Technological Research, VITO NV, Boeretang 200, 2400 Mol, Belgium

**Keywords:** X-ray diffraction computed tomography, XRD-CT, chemical tomography, hyperspectral tomography

## Abstract

An X-ray diffraction computed tomography data-collection strategy that allows, post experiment, a choice between temporal and spatial resolution is reported. This strategy enables time-resolved studies on comparatively short timescales, or alternatively allows for improved spatial resolution if the system under study, or components within it, appear to be unchanging. The application of the method for studying an Mn–Na–W/SiO_2_ fixed-bed reactor *in situ* is demonstrated. Additionally, the opportunities to improve the data-collection strategy further, enabling post-collection tuning between statistical, temporal and spatial resolutions, are discussed. In principle, the interlaced scanning approach can also be applied to other pencil-beam tomographic techniques, like X-ray fluorescence computed tomography, X-ray absorption fine structure computed tomography, pair distribution function computed tomography and tomographic scanning transmission X-ray microscopy.

## Abstract 2

A new data-collection strategy for X­ray diffraction computed tomography experiments is presented that allows, post experiment, a choice between temporal and spatial resolution.

## 1. Introduction   

X-ray diffraction computed tomography (XRD-CT) is an emerging technique that can provide spatially resolved physico-chemical information from within the interiors of intact objects. Originally demonstrated as a laboratory technique, this method has been most usefully applied when using synchrotron X-ray radiation coupled with large-area two-dimensional detectors (Harding *et al.*, 1987 ▸; Bleuet *et al.*, 2008 ▸). Indeed, in the past decade, synchrotron XRD-CT has been employed in several studies and has proven to be a powerful characterization tool to investigate a wide range of inhomogeneous materials (Stock *et al.*, 2008 ▸; Álvarez-Murga *et al.*, 2011 ▸; De Nolf & Janssens, 2010 ▸; Basile *et al.*, 2010 ▸; Artioli *et al.*, 2010 ▸; Palancher *et al.*, 2011 ▸; Valentini *et al.*, 2011 ▸, 2012 ▸; Stock & Almer, 2012 ▸; Voltolini *et al.*, 2013 ▸; Egan *et al.*, 2013 ▸; Ruiz-Martínez *et al.*, 2013 ▸; Bonnin *et al.*, 2014 ▸; Cedola *et al.*, 2014 ▸; Jensen *et al.*, 2015 ▸; Vanmeert *et al.*, 2015 ▸; Wragg *et al.*, 2015 ▸). Furthermore, the nature of the X-rays generated by third-generation synchrotrons (*i.e.* high intensity and brilliance) allows for relatively fast acquisition times, enabling dynamic XRD-CT experiments (Jacques *et al.*, 2011 ▸; O’Brien *et al.*, 2012 ▸; Beale, Gibson *et al.*, 2014 ▸; Price *et al.*, 2015 ▸; Vamvakeros, Jacques, Middelkoop *et al.*, 2015 ▸). In the field of heterogeneous catalysis, such dynamic tomographic experiments are of great importance, as catalytic solids can evolve under operating conditions (Grunwaldt *et al.*, 2013 ▸; Beale, Jacques *et al.*, 2014 ▸).

## 2. X-ray diffraction computed tomography   

As the name suggests, the XRD-CT technique is a marriage of powder X-ray diffraction (PXRD) with computed tomography (CT). Similarly to the first incarnation of traditional X-ray computed tomography (X-ray CT), a pencil-beam scanning approach is used but, instead of recording the absorption of X-rays, the diffraction signal is collected to yield a diffraction projection data set (Hounsfield, 1973 ▸; Elliott & Dover, 1982 ▸). More specifically, the sample is translated along an axis which is perpendicular to the beam axis while being illuminated with a highly collimated or focused monochromatic X-ray beam, and the scattered X-rays are recorded with an area detector (for best counting statistics/speed). In most cases, the size of the translational scan is chosen to be larger than the sample size to ensure that the whole sample is scanned for all measured angles, while the size of the translation step is typically chosen to be the same as the horizontal size of the illuminating X-ray beam. Ideally, as in the case of every pencil-beam scanning tomographic technique, the number of angles measured should be equal to the number of translation steps times π/2 (the Nyquist sampling theorem), but in practice this number can be decreased without significant loss of quality in the reconstructed images and typically the angular range covered is from 0 to π (Álvarez-Murga *et al.*, 2012 ▸).

**Figure 1:**
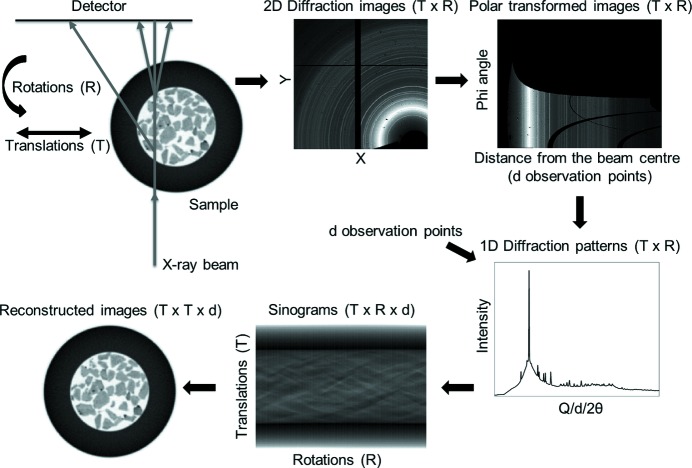
The sample is translated along an axis which is perpendicular to the beam axis while being illuminated with an X-ray beam and rotated, typically covering an angular range of 0 to π, while two-dimensional diffraction patterns (size *X* × *Y*) are recorded with an area detector. The total number of two-dimensional diffraction patterns collected is equal to *T* × *R*, where *T* is the number of translation steps and *R* is the number of rotation steps. The two-dimensional diffraction patterns are then azimuthally integrated to give one-dimensional diffraction patterns (containing *d* observation points) which are plotted as a function of translations and rotations, yielding the sinogram volume (*T* × *R* × *d* matrix). The reconstructed volume (*T* × *T* × *d* matrix) is derived by applying a tomographic reconstruction algorithm to the sinogram volume. The images shown in this figure were derived from the XRD-CT data presented later in this paper.

As shown in Fig. 1 ▸, the raw XRD-CT data collected from a single tomographic scan can be interpreted as an *X* × *Y* × *R* × *T* matrix (*i.e.* a four-dimensional matrix), where *X* × *Y* is the size of the acquired two-dimensional diffraction images, *T* is the number of translation steps and *R* is the number of rotation steps. After performing azimuthal integration of the two-dimensional diffraction images, the size of the matrix is reduced to *R* × *T* × *d* (*i.e.* a three-dimensional matrix), where *d* is the number of observation points in the derived one-dimensional diffraction patterns. The sinograms (*i.e.* the projection data) therefore represent a volume, similar to the case of traditional X-ray CT, but in the case of XRD-CT data the third dimension is not spatial but spectral. If three-dimensional XRD-CT is performed (by acquiring multiple XRD-CT data sets at different positions along the third spatial dimension), then the projection data are stored as a four-dimensional matrix (three spatial dimensions and one spectral). Five-dimensional diffraction imaging can also be achieved by performing successive three-dimensional XRD-CT scans (Beale, Jacques *et al.*, 2014 ▸). In this case, the solid-state changes taking place during the experiment are monitored as a function of time, pressure or temperature, and the data are stored as a five-dimensional matrix. Finally, the reconstructed real-space images are obtained by applying tomographic reconstruction algorithms (*e.g.* algebraic reconstruction techniques or filtered back-projection algorithms) to the projection data (Gordon *et al.*, 1970 ▸; Kak, 1979 ▸; Beister *et al.*, 2012 ▸; Liu, 2014 ▸). An option is to perform peak fitting in the projection data and reconstruct features that contain physical or chemical information (*e.g.* phase distribution maps). However, one has to be careful about this approach as there can be areas in the sample where a specific phase is nanocrystalline, therefore generating very broad diffraction peaks, while in other areas the same phase may be highly crystalline, leading to the formation of very sharp diffraction peaks. In such a case, the peaks should probably be treated as a two-phase problem, otherwise it is impossible to apply a correct peak-shape function to fit the data. This can be easily understood by considering the fact that the sum of two Gaussian functions is not a Gaussian function. However, if there is a distribution of crystallite sizes, then the peak-fitting process becomes more challenging. An alternative option to obtain the reconstructed images is the *reverse analysis* method where the whole projection data set volume is reconstructed, leading to a *T* × *T* × *d* matrix (*i.e.* a three-dimensional matrix). In this case, every pixel in the reconstructed XRD-CT image contains or corresponds to a single diffraction pattern (Bleuet *et al.*, 2008 ▸).

In dynamic XRD-CT, a number of collections are carried out to yield a series of XRD-CT slices showing the spatial changes in chemistry or physical form within a sample over time. To date, the spatial and temporal resolutions in such experiments have been fixed during collection by the choice of acquisition parameters and these resolutions were traded off against one another. High spatial resolution could be obtained but with low temporal resolution and *vice versa*. The risk here is that chemical or physical changes could occur during the collection of a single XRD-CT slice, yielding only a partial understanding of the relationship between spatial composition and time.

### 2.1. Data-collection strategies   

In this section, a review of the existing data-collection strategies to perform an XRD-CT scan is provided (Table 1 ▸) and the advantages and disadvantages of each method are discussed in detail. The main aim of this short review is to find the scanning approach(es) that minimize the dead time in an XRD-CT scan. This dead time is mainly associated with mechanical movement (*i.e.* how the sample moves during the tomographic scan). Optimizing sample movement is essential, as it can lead to a significant decrease in the overall acquisition time of an XRD-CT scan.

#### 2.1.1. Stepped scans   

In the simplest approach, the sample is traversed in fixed steps across the beam and diffraction patterns are collected at each step. The process is then repeated at a number of fixed sample angular rotations, typically covering an angular range from 0 to π. However, this approach is slow and alternative scanning strategies should be used. The reduction of the time required to perform an XRD-CT scan depends not only on the properties of the X-ray beam and the efficiency of the detector, but also on the data-collection strategy. Optimization of the XRD-CT data-collection process is essential, as it not only leads to more efficient use of beamtime but may also be highly desirable for specific experiments. An example of the latter case is the application of XRD-CT to track the evolving solid-state chemistry of functional materials. In such dynamic XRD-CT experiments, the solid-state changes taking place in the sample can take less time than the overall tomographic scan.

#### 2.1.2. Continuous scans   

In another approach, the sample is traversed continuously across the beam and diffraction patterns are collected at a fixed interval, with the process then being repeated at a number of fixed angular rotations (a step angular scan). This collection strategy can significantly reduce the time required to perform a single XRD-CT scan. A similar approach is a continuous angular scan. In this case, the sample is rotated continuously and diffraction patterns are collected at a fixed interval, with the process then repeated at a number of fixed sample traverse steps. The object is rotated at fixed speed about the tomographic axis and diffraction patterns are accumulated over a fixed angular range. This scanning approach requires continuous rotation in the range from 0 to ∼π for each translation. The continuous angular scan approach should be the preferred option, as a much greater portion of reciprocal space is collected compared with the continuous traverse scan approach. More specifically, the crystallites are swept into the diffracting volume, so their orientations are constantly changed and all angles are sampled equally with respect to the axis of rotation. This is important, as the formation of outliers and spots in the raw two-dimensional diffraction images generated by large crystallites can potentially be mitigated. This is a frequently encountered problem in XRD-CT experiments, as such single-crystal artefacts (spots) in the raw two-dimensional diffraction images lead to hotspots in the sinograms and streak artefacts in the reconstructed images (yielding distorted XRD-CT images). The continuous angular scan approach does not guarantee the eradication of single-crystal artefacts but there are strategies available to remove them during the processing of the collected data, post experiment, by applying appropriate filters to the raw two-dimensional diffraction images (Vamvakeros, Jacques, Di Michiel *et al.*, 2015 ▸).

#### 2.1.3. Infinite continuous rotation scan   

An alternative collection strategy is the infinite continuous rotation approach. It is identical to the continuous angular scan approach but in this case the sample is rotated from 0 to *c*π instead of 0 to π, where *c* is a large integer number. More specifically, the new angular position of the sample after every traverse scan is not 0 but *c*π. For example, the angular position is π after the first traverse scan, 2π after the second, 3π after the third *etc*. This means that the dead time of the tomographic scan is reduced, as the sample does not need to be rotated back to 0 after every traverse scan. However, it may not always be experimentally feasible to implement the infinite continuous rotation approach. For example, *in situ* catalytic experiments require gas lines, so more sophisticated reactor cells would be needed in order to use the infinite continuous rotation approach (*e.g.* a reactor in which the gas connections allow for free rotation).

#### 2.1.4. Zigzag scan   

**Figure 2:**
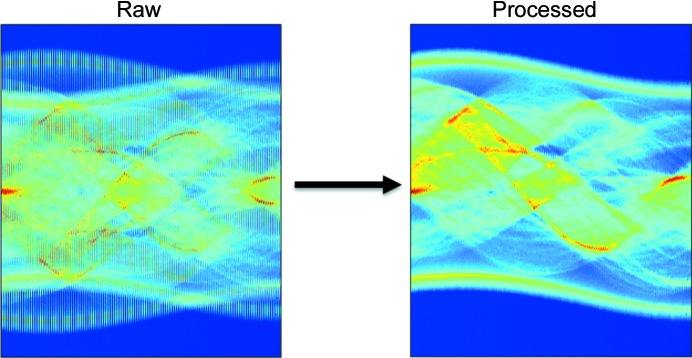
Global sinograms of a 2%La–2%Mn–1.6%Na–3.1%W/SiO_2_ catalyst collected during an XRD-CT scan under ambient conditions using a 46 keV monochromatic pencil beam with a spot size of 2.5 × 2.5 µm. (Left) Raw data using the zigzag approach. (Right) The new global sinogram after sorting the values appropriately.

A zigzag scanning approach can also significantly reduce the dead time of the tomographic measurement without the need for specially designed reactors (in contrast to the infinite continuous rotation approach). The zigzag approach can easily be combined with all the previously mentioned collection strategies (*i.e.* stepped and continuous scans). For example, when the continuous traverse scan is used, the sample is returned to the initial position after every angular step. It is possible to avoid the dead time associated with this movement by performing the scan at the next angle but setting the new starting position (for the traverse scan) as the final position of the previous scan (*i.e.* the starting positions of the sample will be alternately 0 and π for every traverse step). As expected, the values in the three-dimensional matrix of the projection data (the sinograms) need to be sorted appropriately before a reconstruction tomographic algorithm is used. This is shown in Fig. 2 ▸, where a global sinogram from a continuous traverse zigzag XRD-CT scan (left) and the new sinogram after sorting the values appropriately (right) are shown.

## 3. Interlaced X-ray diffraction computed tomography   

In this study, we report a new data-collection strategy, interlaced XRD-CT (IXRD-CT), which allows one, post experiment, to choose between temporal and spatial resolution. In a typical XRD-CT experiment, the number of translation and rotation steps (or equivalently the total length and the angular range to be covered) and the values of these steps define the spatial and temporal resolution of the tomographic measurement. Unfortunately, although both high spatial and high temporal resolution are desired, there is always a trade-off between the two when conventional data-collection strategies are employed. This means that high spatial resolution scans have low temporal resolution, while high temporal resolution scans have low spatial resolution. IXRD-CT offers a way of bridging this gap by providing temporal resolution inside a high spatial resolution XRD-CT scan. The basic principle of this method consists of performing subsequent XRD-CT scans with low spatial but high temporal resolution, which can then be easily combined, post experiment, to yield the same results as the equivalent high spatial resolution XRD-CT scan.

In an XRD-CT scan, the spatial resolution is defined by the traverse step size which is typically, but not necessarily, the same as the horizontal size of the illuminating X-ray beam. In an IXRD-CT scan, the time resolution is chosen before the tomographic measurement begins. There are two types of IXRD-CT scan: (i) the continuous traverse IXRD-CT scan and (ii) the continuous angular IXRD-CT scan. Both methods are explained in the following sections. The work presented herein demonstrates the feasibility of performing IXRD-CT scans and the advantages of applying such a scanning approach in XRD-CT experiments. However, it should be highlighted that the interlaced scanning approach can, in principle, be applied to other pencil-beam chemical (*i.e.* hyperspectral) tomographic techniques, like X-ray fluorescence computed tomography (XRF-CT), X-ray absorption fine structure computed tomography (XAFS-CT), pair distribution function computed tomography (PDF-CT) (Jacques *et al.*, 2013 ▸) and tomographic scanning transmission X-ray microscopy (STXM) (Boisseau, 1986 ▸; Boisseau & Grodzins, 1987 ▸; Schroer *et al.*, 2003 ▸; Wang *et al.*, 2000 ▸; Johansson *et al.*, 2007 ▸).

### 3.1. Continuous traverse IXRD-CT scan   

In the case of the continuous traverse IXRD-CT scan, the value of the angular step size (*S*_a_) corresponding to a desired high spatial resolution XRD-CT scan is initially chosen (*e.g.* an angular step size of 1.5°). The next step is to choose the temporal resolution (*R*_t_) of the IXRD-CT scan. This is defined as an integer value which represents how many times the temporal resolution is increased compared with the high spatial resolution XRD-CT scan. For example, if the value of *R*_t_ is 8, then the temporal resolution of the IXRD-CT scan will be eight times higher than the high spatial resolution XRD-CT scan. The IXRD-CT scan will therefore consist of *R*_t_ XRD-CT scans with an angular step size of *S*_a_ × *R*_t_. For example, if *S*_a_ is 1.5° and *R*_t_ is 8, then the new angular step size will be 12°.

**Figure 3:**
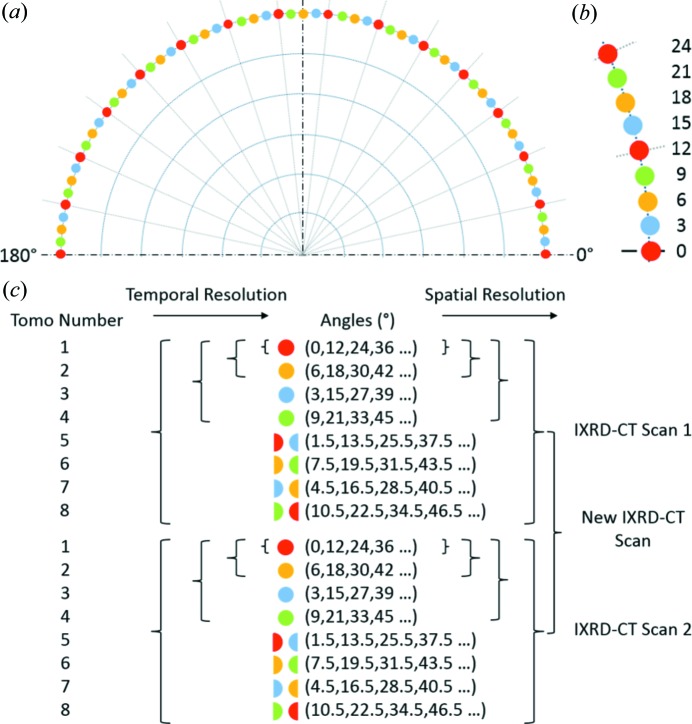
(*a*) A schematic representation of a continuous traverse IXRD-CT scan consisting of eight XRD-CT scans. (*b*) An expanded section of part (*a*), showing the angular positions of the individual XRD-CT scans. (*c*) A demonstration of how the spatiotemporal resolution changes in the IXRD-CT scan and the possible combinations of individual data sets.

As mentioned previously, the angular range typically covered in an XRD-CT scan is 0–180°. The *R*_t_ XRD-CT scans composing the IXRD-CT scan cover the same angular range (*i.e.* 180° in total) and have the same angular step size but the starting angular positions are different. These angles can be calculated easily: 0, *S*_a_, 2*S*_a_, 3*S*_a_, …, (*R*_t_ − 1)*S*_a_. An example, where *S*_a_ is 1.5° and *R*_t_ is 8, is provided in Fig. 3 ▸. The starting angles of the eight tomographic scans are 0, 1.5, 3, 4.5, 6, 7.5, 9 and 10.5°. As can be readily understood, combining these eight tomographic data sets, post experiment, will give results identical to a high spatial resolution XRD-CT scan of 1.5° angular step size covering an angular range of 0 to 180°. At the same time, each one of the *R*_t_ XRD-CT scans has *R*_t_ times lower spatial and *R*_t_ times higher temporal resolution compared with an IXRD-CT scan.

The final step is to decide the order of the *R*_t_ XRD-CT scans. This step is crucial and should be not treated lightly, as it determines whether the temporal resolution will be directly linked to the spatial one or not. In the case of the previously mentioned example, the optimal order of the individual *R*_t_ XRD-CT scans is shown in Fig. 3 ▸(*c*). These *R*_t_ tomographic scans are referred to as ‘tomo’ numbers in Fig. 3 ▸ (*i.e.* tomo numbers 1 to 8). Furthermore, combining tomo pairs *k* and *k* + 1, where *k* is 1, 3, 5 and 7 (*i.e.* tomo numbers 1 and 2, 3 and 4, 5 and 6, and 7 and 8), leads to XRD-CT data sets with four times higher temporal resolution and four times lower spatial resolution compared with the complete IXRD-CT scan. Similarly, combining tomo pairs *k* to *k* + 3, where *k* is 1 and 5 (*i.e.* tomo numbers 1–4 and 5–8), leads to XRD-CT data sets with two times higher temporal resolution and two times lower spatial resolution compared with the complete IXRD-CT scan. It should also be noted that the results from successive IXRD-CT scans can be combined to yield new data sets [*e.g.* the new IXRD-CT scan shown in Fig. 3 ▸(*c*)]. This could be essential in a dynamic experiment where solid-state changes can take place during a single tomographic scan.

**Figure 4:**
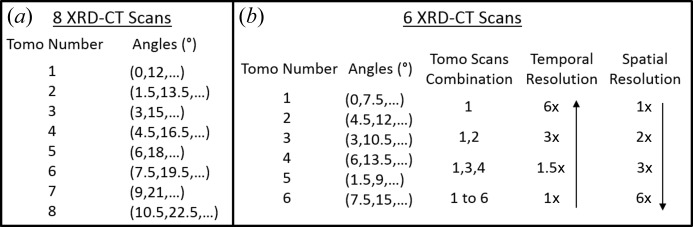
(*a*) A potential order for performing an IXRD-CT scan consisting of eight XRD-CT scans. (*b*) A demonstration of how the spatiotemporal resolution changes in an IXRD-CT scan consisting of six XRD-CT scans. The possible combinations of individual data sets are presented.

However, if the order of the eight XRD-CT scans composing the complete IXRD-CT scan is different from those previously mentioned, then the spatial and temporal resolutions cannot be linked appropriately. For example, an alternative order for performing these scans is presented in Fig. 4 ▸(*a*). In this case, half the spatial resolution (*i.e.* by combining tomo scans 1, 3, 5 and 7) does not lead to double the temporal resolution but to 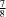 of the temporal resolution of the complete IXRD-CT scan. Similarly, 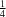 of the spatial resolution (*i.e.* by combining tomo scans 1 and 5) does not lead to a four times higher temporal resolution but only 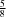 of the complete IXRD-CT scan. As a result, the full potential of this IXRD-CT measurement (eight XRD-CT scans with an angular step of 12°) is not reached.

At this point, it should be noted that the number *R*_t_ of tomographic scans composing the IXRD-CT scan should not be an odd number as the same problem arises. Although any even number can be used, ideally *R*_t_ should be a power of 2 (*e.g.* 2, 4 and 8). In Fig. 4 ▸(*b*), an example is given when the IXRD-CT scan consists of six XRD-CT scans. In this case, the angular step is 9° and the optimal order for performing the individual scans is shown in Fig. 4 ▸(*b*). However, it can be seen that the spatial and temporal resolutions cannot be perfectly linked even when the optimal order is chosen. More specifically, combining tomo scans 1, 3 and 4 gives half the spatial but 1.5 times the temporal resolution of the complete IXRD-CT scan (instead of double). This happens because the time to perform tomo scan 2 has to be taken into account too.

### 3.2. Continuous angular IXRD-CT scan   

The basic principle of the continuous angular IXRD-CT scan is the same as the continuous traverse IXRD-CT scan. The only difference is that now the fast tomographic axis is the rotation axis and the slow tomographic axis is the translation axis. For example, if the slow-axis step size (*i.e.* the translational step) is a number *l* corresponding to a specific length (*e.g.* the horizontal size of the X-ray beam) and *R*_t_ is chosen to be 8, then the spatial and temporal resolutions of the complete IXRD-CT scan will be identical to the previous continuous traverse IXRD-CT example. More specifically, in both types of IXRD-CT, the angular range covered is 0 to 180° with a step of 1.5° and the same length is covered with a step of *l*. However, the sinograms change in a different way. In the case of a continuous traverse IXRD-CT scan, as will be shown later, combining different XRD-CT scans increases the number of limits of the axis corresponding to rotations. For clarity, the XRD-CT scans composing the complete IXRD-CT scan will be referred to as tomo scans in the rest of this paper.

### 3.3. Demonstration and comparison of the IXRD-CT methods   

**Figure 5:**
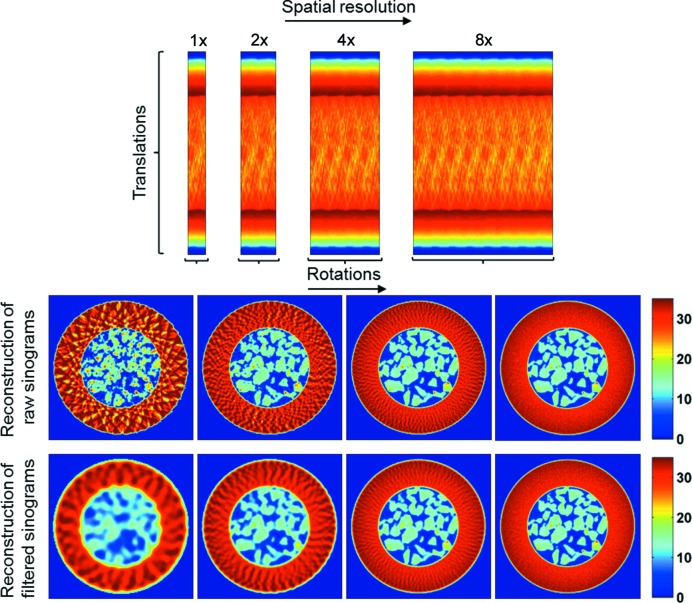
Continuous traverse IXRD-CT scan: the spatial resolution can be increased by combining different XRD-CT scans. The limits of the sinogram axis corresponding to translations remain constant, while the limits of the sinogram axis corresponding to rotations gradually increase, increasing the spatial resolution of the respective reconstructed images.

A demonstration using the previous continuous traverse IXRD-CT example is provided in Fig. 5 ▸, where the presented sinogram corresponds to the global sinogram (*i.e.* the sinogram volume summed along the third dimension, which is the spectral dimension) of an IXRD-CT experiment of a fixed-bed reactor. More details about the experiment are provided in the next section. The filtered back-projection algorithm (Kak & Slaney, 1988 ▸) was chosen to reconstruct the sinograms as it is very fast and easy to implement. It can be seen in Fig. 5 ▸ that the direct reconstruction of the sinograms leads to artefacts in the reconstructed images. This is apparent in the high temporal/low spatial resolution scan (*i.e.* 1× in Fig. 5 ▸) where there are intensity variations present in the reconstructed images, suggesting that the sample is highly inhomogeneous. Furthermore, there are regions in the images where the intensity is higher than the background, implying that the sample is present in these areas. However, both these phenomena are artefacts generated because of the angular undersampling (Fig. S1 in the supporting information). Artefacts due to undersampling (*i.e.* the limited number of projections) are a well known problem in traditional X-ray CT (Kak & Slaney, 1988 ▸). Herein, these artefacts have been mitigated by convoluting the sinograms with an appropriate window function prior to reconstruction (by changing the width of the Hann window accordingly). As expected, reconstructing the filtered sinograms leads to the lower spatial resolution images shown in Fig. 5 ▸ but the previously mentioned artefacts have been removed. There are options one can explore in order to optimize the quality of the reconstructed images of the high temporal resolution tomo scans. For example, the effect of different reconstruction algorithms can be investigated (Figs. S2 and S3 in the supporting information). However, this is beyond the scope of the work presented here.

**Figure 6:**
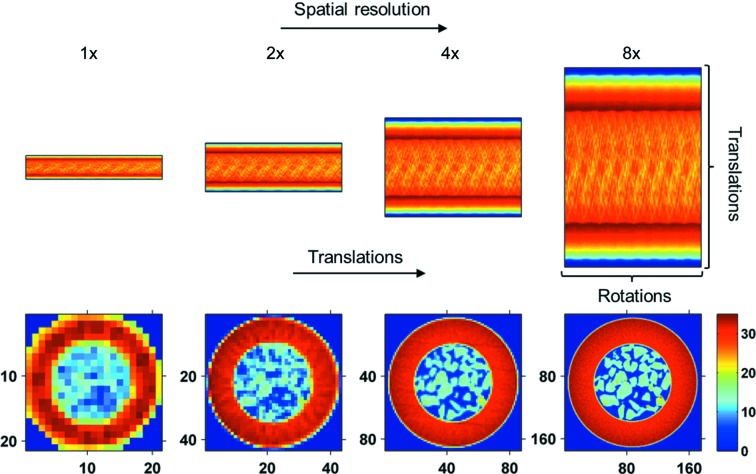
Continuous angular IXRD-CT scan: the spatial resolution can be increased by combining different XRD-CT scans. The limits of the sinogram axis corresponding to the angles sampled remain constant, while the limits of the sinogram axis corresponding to translations increase gradually, increasing not just the spatial resolution but also the size of the reconstructed images.

In contrast, in the case of the continuous angular IXRD-CT method it is the sinogram axis corresponding to translations that changes, while the sinogram axis corresponding to rotations remains the same. As a result, in a continuous angular IXRD-CT scan the resolution of the reconstructed XRD-CT images changes during the IXRD-CT. This is clearly shown in the simulation presented in Fig. 6 ▸ (using the complete sinogram presented in Fig. 5 ▸), where the sinograms and the corresponding reconstructed images are presented. In a continuous angular IXRD-CT scan, the traverse step size is several times larger than the horizontal size of the illuminating X-ray beam, meaning that there are parts of the sample that are not scanned, leading to loss of information. Furthermore, should a problem occur during acquisition of the tomo scans (*e.g.* beam refill, significant intensity variations of the X-ray beam), the sinograms cannot easily be corrected by applying a simple scale factor as in the case of the continuous traverse IXRD-CT scan (*i.e.* if there are no solid-state changes during acquisition, then the total scattering intensity for every line scan should be the same). In summary, the problems that need to be addressed with a continuous angular IXRD-CT scan can be summarized as the following: (i) the sinograms of the corresponding tomo scans have to be independently centred; (ii) the size of the reconstructed images changes when sinograms from different tomo scans are combined; (iii) there is a potential loss of information because the whole sample is not scanned in every tomo scan, and this information requires further data processing to be retrieved (if possible); and (iv) the requirement for a stable X-ray beam. Taking into account the previous reasons and the increased complexity of data acquisition and processing without evident gain in information or image quality, it was decided to perform the present experiments using the continuous traverse IXRD-CT approach.

## 4. Proof of concept   

A fixed-bed reactor consisting of a 2%Mn–1.6%Na–3.1%W/SiO_2_ catalyst bed supported with glass wool was tested at station ID15A of the ESRF. Details of the preparation of the catalyst are given in the supporting information. The reactor was mounted into a gas-delivery stub, itself mounted on a standard goniometer. The goniometer was fixed to a rotation stage set upon a translation stage to facilitate the movements required for the CT measurement. Heating was achieved by virtue of two hot-air blowers heating each side of the catalytic membrane reactor. A state-of-the-art PILATUS3X CdTe 300K hybrid photon-counting area detector, which uses cadmium telluride (CdTe) as the semiconducting direct-conversion layer, was used to record the two-dimensional diffraction patterns. The acquisition time per point was 50 ms. Tomographic reconstruction was performed using filtered back-projection. IXRD-CT measurements were performed using a 93 keV monochromatic pencil beam with a spot size of 25 × 25 µm. The IXRD-CT measurements were made with 180 translations over 180° in 1.5° steps covering a physical area of 4.5 × 4.5 mm. Reconstruction of these data yielded images of 180 × 180 pixels and 25 µm resolution. Each IXRD-CT scan consisted of eight XRD-CT scans. The angular step of the individual XRD-CT scans was 12° and the order of these scans was the same as that presented in Fig. 3 ▸. Copies of the radially integrated XRD-CT data can be found at http://tiny.cc/C5CC03208C.

The data-collection strategy used in this tomographic experiment is the continuous traverse IXRD-CT scanning approach. The results from two successive IXRD-CT scans of the 2%Mn–1.6%Na–3.1%W/SiO_2_ catalyst during temperature ramping from 728 to 1038 K with a ramp rate of 4.5 K min^−1^ under He flow (30 ml min^−1^) are presented in this section. In the interest of brevity, the two complete IXRD-CT scans will be referred to as IXRD-CT scan 1 and IXRD-CT scan 2. Each complete IXRD-CT scan is composed of eight XRD-CT scans which will be referred to as tomo scans 1 to 8. We have recently reported that the main crystalline phases present in this catalyst under ambient conditions are cristobalite, tridymite (both SiO_2_ polymorphs), Mn_2_O_3_ and Na_2_WO_4_ (Vamvakeros, Jacques, Middelkoop *et al.*, 2015 ▸). This catalyst is well established for the oxidative coupling of methane (OCM) to produce ethylene (Arndt *et al.*, 2012 ▸). The melting point of Na_2_WO_4_ in 1 bar pressure (1 bar = 100 000 Pa) is 968 K, which is significantly lower than the temperature required for the OCM reaction (Haynes, 2014 ▸; Goranson & Kracek, 1935 ▸). This means that Na_2_WO_4_ is expected to be present in a molten state under OCM conditions (Sadjadi *et al.*, 2015 ▸; Vamvakeros, Jacques, Middelkoop *et al.*, 2015 ▸). Therefore, this catalyst was considered to be an ideal system to test the feasibility of IXRD-CT measurements as there are solid-state changes taking place during temperature ramping, even under the flow of inert gases. Herein, we will be mainly focusing on the evolution of the Na_2_WO_4_ phase.

### 4.1. Space series   

**Figure 7:**
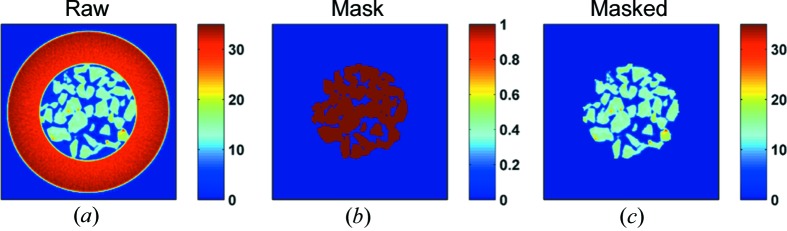
(*a*) The reconstructed image of the global sinogram of IXRD-CT scan 1 shown in Fig. 5 ▸. (*b*) The mask created to separate the catalyst particles from the capillary (glassware). (*c*) The reconstructed image after applying the mask.

An appropriate mask, as shown in Fig. 7 ▸, has been applied to all the reconstructed images presented in this work in order to remove the contribution from the capillary and show only the sample of interest. This mask was created using the reconstructed image of the global sinogram shown in Fig. 5 ▸. Also in the figures, where parts of the complete IXRD-CT sinograms are used (*i.e.* less than eight tomo scans), the respective sinograms were convoluted with an appropriate Hann window function prior to reconstruction, as discussed previously.

**Figure 8:**
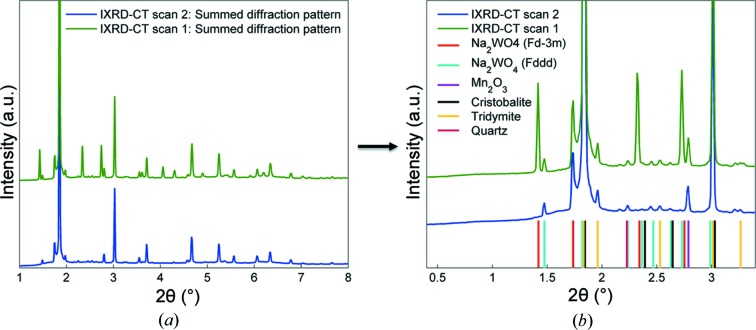
(*a*) Summed diffraction patterns for IXRD-CT scans 1 and 2. (*b*) A region of interest of the two diffraction patterns, showing the main diffraction peaks generated by the two Na_2_WO_4_ phases, Mn_2_O_3_, cristobalite, tridymite and quartz.

Fig. 8 ▸ presents the summed diffraction patterns of the two IXRD-CT scans (summing the reconstructed volume along the two spatial dimensions). Also shown are the main diffraction peaks generated by two crystalline SiO_2_ polymorphs (*i.e.* cristobalite and tridymite), Mn_2_O_3_ and two Na_2_WO_4_ phases. Minor peaks corresponding to SiO_2_ quartz were also identified. It can clearly be seen that the peaks corresponding to the high-symmetry (cubic) Na_2_WO_4_ phase are not present in the IXRD-CT scan 2 diffractogram. The transformation of the cubic Na_2_WO_4_ phase to a lower-symmetry orthorhombic Na_2_WO_4_ phase will be discussed in more detail in the following section.

In the 1930s, it was suggested that two phase transitions of anhydrous Na_2_WO_4_ (phase I) take place above 858 K, the first (phase II) being stable for only a few kelvin and the second (phase III) being stable up to the melting point of Na_2_WO_4_ (Goranson & Kracek, 1935 ▸; Austin & Pierce, 1935 ▸). Later studies showed that the high-symmetry cubic (space group 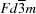) phase I of Na_2_WO_4_ changes to the lower-symmetry phase III and the orthorhombic *Pnam* space group was suggested after peak indexing (Pistorius, 1966 ▸). However, it has to be noted that the high-temperature PXRD measurement was performed at 901 K, which is significantly lower than the melting point of Na_2_WO_4_. Further studies of the binary Na_2_WO_4_–Na_2_MoO_4_ system by Bottelberghs & van Buren (1975 ▸) suggested that different structural changes take place: cubic 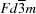 to orthorhombic *Pbn*2_1_ at 861 K, *Pbn*2_1_ to ortho­rhombic *Fddd* at 863 K and *Fddd* to hexagonal *P*6_3_/*mmc* above 913 K. Recent high-temperature Raman studies showed that there is a transition from the high-symmetry cubic Na_2_WO_4_ phase to a lower symmetry above 833 K (Lima *et al.*, 2011 ▸). In that study, the Na_2_WO_4_ system was investigated up to 918 K. To the best of our knowledge, there are no high-temperature PXRD studies in the literature showing a crystalline Na_2_WO_4_ phase being present above 923 K. More specifically, two high-temperature PXRD studies of an NaWO_4_-containing catalyst (*i.e.* an Mn–Na–W/SiO_2_ and an NaCl–Mn–Na–W/SiO_2_ catalyst, respectively) have shown that there are no crystalline Na_2_WO_4_ or other W-containing phases present above 923 K (Hou *et al.*, 2006 ▸; Hiyoshi & Ikeda, 2015 ▸).

In this experiment, the cubic Na_2_WO_4_ phase disappears completely at approximately 873 K. The new Na_2_WO_4_ phase starts appearing above 838 K, as suggested by Lima *et al.* (2011 ▸), but only one peak can be observed from the diffraction patterns. The summed diffraction patterns for every translational scan for the two IXRD-CT scans (240 in total) are plotted in Fig. S5 of the supporting information. The new phase is apparent above 858 K, where all the peaks are clearly visible, and this phase disappears completely at approximately 933 K. No Na_2_WO_4_ peaks are observed up to the final temperature of 1038 K.

Peak indexing can be very challenging as there are numerous phases present in the catalyst and there are peaks overlapping. For this reason, the space groups suggested by the previous studies were used. The published crystallographic information file after Pistorius (space group *Pnam*, PDF card No. 00-020-1163) does not predict all the peaks associated with the new Na_2_WO_4_ phase (Pistorius, 1966 ▸). Similar results are obtained when the hexagonal *P*6_3_/*mmc* space group is used. However, when the orthorhombic *Fddd* space group is used, all the peaks in the diffractograms are predicted, a symmetry which has been suggested in the literature in the past (Bottelberghs & van Buren, 1975 ▸). This is clearly shown on the right-hand side of Fig. 8 ▸. In Fig. S6 in the supporting information, Pawley whole powder pattern fitting was also performed with the *GSASII* software using the appropriate unit cells to ensure that no peaks were neglected after the phase identification (Toby & Von Dreele, 2013 ▸). The summed diffraction from tomo scan 1 of IXRD-CT scan 2 was used for the Pawley analysis, corresponding to a temperature range of 893–913 K, as there is only the low-symmetry Na_2_WO_4_ phase present and no cubic Na_2_WO_4_.

**Figure 9:**
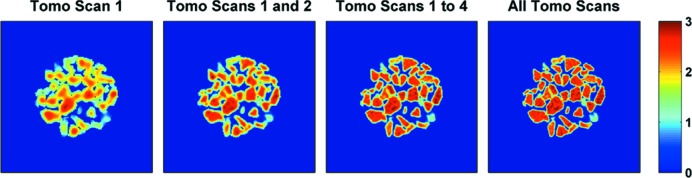
Reconstructed images corresponding to the main diffraction peak of cristobalite (*i.e.* reflection 111) from the IXRD-CT scan 1 data set. It is shown that combining multiple tomo scans leads to a significant increase in spatial resolution.

First, the results from IXRD-CT scan 1 are presented. In Fig. 9 ▸, reconstructed images of cristobalite corresponding to a scattering angle 2θ of 1.85° (reflection 111) are shown. The reconstructed images corresponding to cristobalite define well the shape and size of the catalyst particles as it is the main crystalline phase of the catalyst support. As discussed previously, it is possible to combine the different tomo scans composing the IXRD-CT scan post experiment. Such an example is presented in Fig. 9 ▸, where it is shown how the spatial resolution gradually increases when different tomo scans are combined.

At this point, another major advantage of the IXRD-CT method should be highlighted: the diffraction peaks present in the IXRD-CT data can be treated independently. More specifically, observation points in the reconstructed data corresponding to diffraction peaks generated by a crystalline phase that does not change during an experiment can be accumulated over time (*i.e.* using different or successive IXRD-CT data sets), thus improving the statistics. One may therefore argue that in fact there are not two but three resolutions that can be directly linked in IXRD-CT experiments: spatial, temporal and statistical.

**Figure 10:**
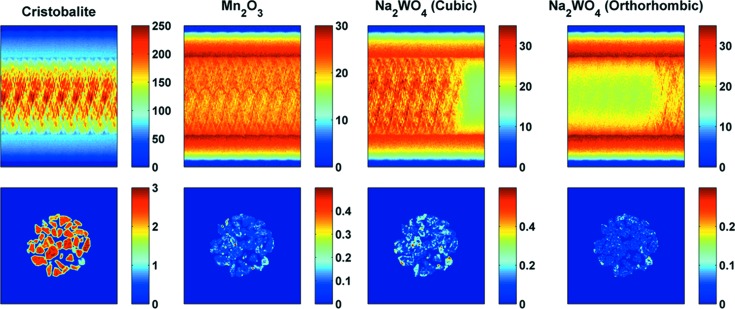
The sinograms from IXRD-CT scan 1 corresponding to cristobalite, Mn_2_O_3_ and two Na_2_WO_4_ phases, and their respective reconstructed images (temperature range 728–883 K). The sinograms correspond to scattering angles 2θ of 1.85, 2.79, 1.42 and 1.48°, respectively.

In Fig. 10 ▸, reconstructed images of cristobalite, Mn_2_O_3_ and two Na_2_WO_4_ phases corresponding to scattering angles 2θ of 1.85 (reflection 111), 2.79 (reflection 222), 1.42 (reflection 111) and 1.48° (reflection 111), respectively, are shown. These 2θ angles correspond to the highest-intensity diffraction peaks generated by these phases. Inspection of the sinograms reveals that there is an Na_2_WO_4_ phase transition taking place during IXRD-CT scan 1. It should be noted here that it is fundamentally wrong to reconstruct the sinograms corresponding to the two Na_2_WO_4_ phases, as the sample is not present in some parts of these two sinograms. Here exactly lies the advantage of an IXRD-CT scan: it allows the tracking of solid-state changes taking place during a tomographic scan, and the reconstruction of the respective sinograms does not violate the principles of computed tomography. This will be demonstrated in the next section. In Fig. 10 ▸, it can also be seen that the Mn_2_O_3_ is not co-located with the cubic Na_2_WO_4_, which is in agreement with our previous study (Vamvakeros, Jacques, Middelkoop *et al.*, 2015 ▸). This is important as it simplifies the interpretation of the results. More specifically, it can be seen in Fig. 10 ▸ that the new phase that appears is co-located with the cubic Na_2_WO_4_ phase, suggesting that this is another Na–W–O phase.

**Figure 11:**
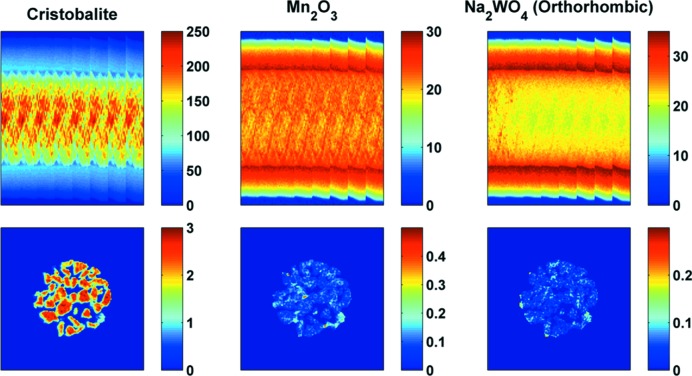
The sinograms from IXRD-CT scan 2 corresponding to cristobalite, Mn_2_O_3_ and Na_2_WO_4_ (low-symmetry) phases, and their respective reconstructed images (temperature range 883–1038 K). The sinograms correspond to scattering angles 2θ of 1.85, 2.79 and 1.48°, respectively.

The results from IXRD-CT scan 2 are presented in Fig. 11 ▸, where the sinograms and the corresponding reconstructed images of cristobalite, Mn_2_O_3_ and Na_2_WO_4_ (orthorhombic) are shown. The cubic Na_2_WO_4_ phase is no longer present and the orthorhombic Na_2_WO_4_ phase disappears at approximately 933 K. No other Na–W–O phases were observed at higher temperatures. This is in agreement with our previous study of a catalytic membrane reactor containing this catalyst (Vamvakeros, Jacques, Middelkoop *et al.*, 2015 ▸).

### 4.2. Time series   

**Figure 12:**
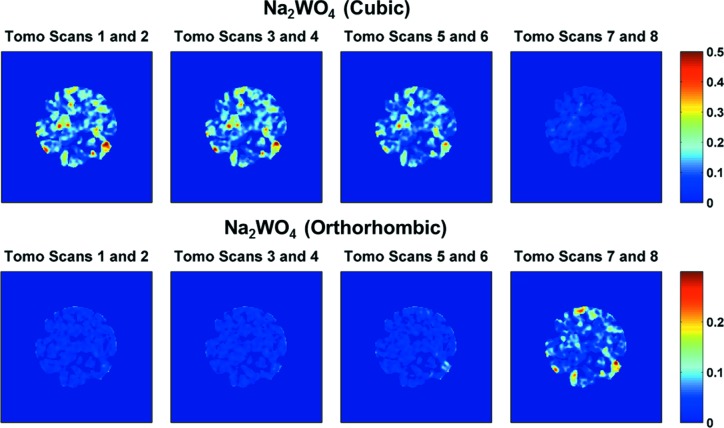
(Top row) Reconstructed images corresponding to the high-symmetry Na_2_WO_4_ phase. (Bottom row) Reconstructed images corresponding to the low-symmetry Na_2_WO_4_ phase. Temperature ranges: 728–767 K for tomo scans 1 and 2, 767–806 K for tomo scans 3 and 4, 806–844 K for tomo scans 5 and 6, and 844–883 K for tomo scans 7 and 8.

An example of the temporal resolution that an IXRD-CT scan can provide is demonstrated in Fig. 12 ▸. The reconstructed images presented correspond to the two Na_2_WO_4_ phases (scattering angles 2θ 1.42 and 1.48°, respectively) present in the catalyst. The acquisition time of the complete IXRD-CT scan was approximately 35 min, and therefore the reconstructed images shown in Fig. 12 ▸ correspond to 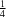 of the overall time (approximately 8.75 min). It can clearly be seen that the orthorhombic Na_2_WO_4_ phase forms/grows when the cubic Na_2_WO_4_ phase disappears.

**Figure 13:**
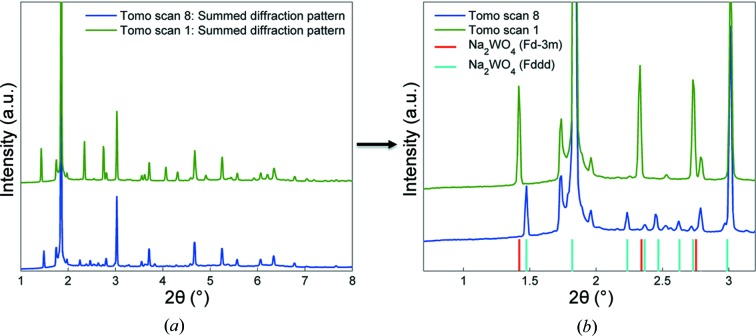
(*a*) Summed diffraction patterns of tomo scans 1 and 8 of IXRD-CT scan 1. (*b*) A region of interest of the two diffraction patterns, showing the main diffraction peaks generated by the two Na_2_WO_4_ phases.

In Fig. 13 ▸, the summed diffraction patterns from two tomo scans (tomo scans 1 and 8) are presented. Both these tomo scans belong to IXRD-CT scan 1. On the right-hand side of Fig. 13 ▸, a region of interest of these two diffraction patterns is selected, showing the high-intensity peaks corresponding to the cubic and orthorhombic Na_2_WO_4_ phases (red and cyan lines, respectively).

**Figure 14:**
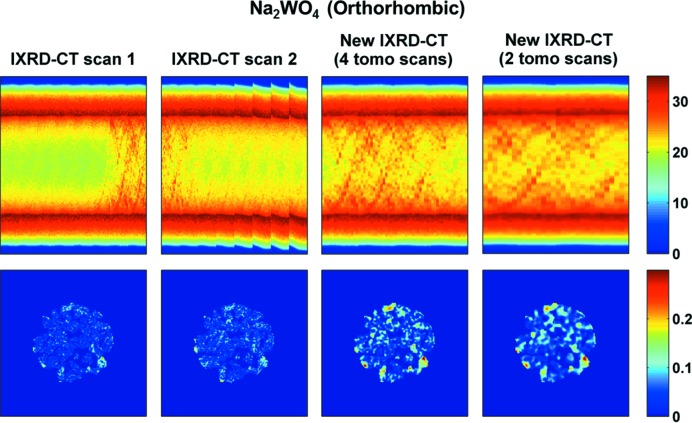
Sinograms and reconstructed images of the orthorhombic Na_2_WO_4_ phase using different IXRD-CT data sets. First column: IXRD-CT scan 1. Second column: IXRD-CT scan 2. Third column: the new IXRD-CT data set derived by combining tomo scans 7 and 8 from IXRD-CT scan 1 and tomo scans 1 and 2 from IXRD-CT scan 2. Fourth column: the new IXRD-CT data set derived by combining tomo scan 8 from IXRD-CT scan 1 and tomo scan 1 from IXRD-CT scan 2.

Finally, as discussed previously, it is possible to combine tomo scans from successive IXRD-CT scans (Fig. 3 ▸). An example is provided in Fig. 14 ▸, where it is shown that by combining tomo scans 7 and 8 from IXRD-CT scan 1 with tomo scans 1 and 2 from IXRD-CT scan 2, a new IXRD-CT data set is created. Another possibility, also shown in Fig. 14 ▸, is to combine tomo scan 8 from IXRD-CT scan 1 and tomo scan 1 from IXRD-CT scan 2. This is important, as it can be seen that the reconstructed image of the new sinogram corresponding to the low-symmetry Na_2_WO_4_ phase yields higher-quality images. In this case, higher quality simply means that it is easier to identify the high-concentration areas of this phase in the catalyst particles compared with the complete IXRD-CT scans.

## 5. Conclusions   

We have presented a short review of the existing data-collection strategies during an XRD-CT measurement and discussed the advantages and disadvantages of each method. A superior data-collection strategy, interlaced XRD-CT (IXRD-CT), has been suggested as a method that allows, post experiment, a choice between temporal and spatial resolution. The main advantages of the IXRD-CT method can be summarized as the following:

(i) High spatial resolution can be chosen when the system is not changing.

(ii) High temporal resolution can be chosen when the system is changing.

(iii) Data from successive XRD-CT scans can be combined.

(iv) Different Bragg reflections can be treated independently.

This method not only enables dynamic XRD-CT studies on comparatively short timescales but also allows for improved spatial resolution if the system under study, or components within it, appear to be unchanging. In this study, the feasibility of performing an IXRD-CT experiment was demonstrated using an OCM catalyst that was studied during temperature ramping under inert conditions. It was shown that increased time resolution can be achieved and solid-state changes taking place inside a complete IXRD-CT scan could be tracked accurately. The real power in XRD-CT experiments is to be found when the technique is employed in time-resolved mode and the interlaced scanning approach is the best method to retain the option of time *versus* spatial resolution without suffering the consequences of an ‘incorrect’ choice when measuring unknown samples whose behaviour may be less predictable. In principle, the interlaced scanning approach can also be applied to other pencil-beam chemical (*i.e.* hyperspectral) tomographic techniques, like XRF-CT, XAFS-CT, PDF-CT and tomographic STXM.

## 6. Related literature   

For additional literature relating to the supporting information, see Pan & Kak (1983 ▸), Andersen & Kak (1984 ▸), Hansen & Saxild-Hansen (2012 ▸), Censor *et al.* (2007 ▸, 2001 ▸), Landweber (1951 ▸) and Cimmino (1938 ▸).

## Supplementary Material

Supporting information file. DOI: 10.1107/S160057671600131X/nb5168sup1.pdf

## Figures and Tables

**Table 1:** Existing XRD-CT collection strategies

Scan strategy	Description
(1) Step traverse scan and stepped angular scan	The sample is traversed in fixed steps across the beam and diffraction patterns are collected at each step, with the process then repeated at a number of fixed sample angular rotations
(2) Step angular scan and stepped traverse scan	As with (1), except the traverse and angle sequence is swapped
(3) Continuous traverse scan and stepped angular scan	The sample is traversed continuously across the beam and diffraction patterns are collected at fixed intervals, with the process then repeated at a number of fixed sample angular rotations
(4) Continuous angular scan and stepped traverse scan	The sample is rotated continuously and diffraction patterns are collected at fixed intervals, with the process then repeated at a number of fixed sample traverse steps
(5) Zigzag scan	The new starting position of the sample at the end of every traverse scan (in the case of a continuous traverse scan) or angular scan (in the case of a continuous angular scan) is the final position of the previous scan

## References

[bb1] Álvarez-Murga, M., Bleuet, P. & Hodeau, J.-L. (2012). *J. Appl. Cryst.* **45**, 1109–1124.

[bb2] Álvarez-Murga, M., Bleuet, P., Marques, L., Lepoittevin, C., Boudet, N., Gabarino, G., Mezouar, M. & Hodeau, J.-L. (2011). *J. Appl. Cryst.* **44**, 163–171.

[bb52] Andersen, A. H. & Kak, A. C. (1984). *Ultrason. Imaging*, **6**, 81–94. 10.1177/016173468400600107 6548059

[bb3] Arndt, S., Otremba, T., Simon, U., Yildiz, M., Schubert, H. & Schomäcker, R. (2012). *Appl. Catal. Gen.* **425–426**, 53–61.

[bb4] Artioli, G., Cerulli, T., Cruciani, G., Dalconi, M. C., Ferrari, G., Parisatto, M., Rack, A. & Tucoulou, R. (2010). *Anal. Bioanal. Chem.* **397**, 2131–2136. 10.1007/s00216-010-3649-0 20358183

[bb5] Austin, J. B. & Pierce, R. H. H. Jr (1935). *J. Chem. Phys.* **3**, 683–686.

[bb6] Basile, F., Benito, P., Bugani, S., De Nolf, W., Fornasari, G., Janssens, K., Morselli, L., Scavetta, E., Tonelli, D. & Vaccari, A. (2010). *Adv. Funct. Mater.* **20**, 4117–4126.

[bb7] Beale, A. M., Gibson, E. K., O’Brien, M. G., Jacques, S. D. M., Cernik, R. J., Michiel, M. D., Cobden, P. D., Pirgon-Galin, Ö., van de Water, L., Watson, M. J. & Weckhuysen, B. M. (2014). *J. Catal.* **314**, 94–100.

[bb8] Beale, A. M., Jacques, S. D. M., Gibson, E. K. & Di Michiel, M. (2014). *Coord. Chem. Rev.* **277–278**, 208–223.

[bb9] Beister, M., Kolditz, D. & Kalender, W. A. (2012). *Phys. Med.* **28**, 94–108. 10.1016/j.ejmp.2012.01.003 22316498

[bb10] Bleuet, P., Welcomme, E., Dooryhée, E., Susini, J., Hodeau, J. L. & Walter, P. (2008). *Nat. Mater.* **7**, 468–472. 10.1038/nmat2168 18425135

[bb11] Boisseau, P. (1986). PhD thesis. Department of Physics, Massachusetts Institute of Technology, Cambridge, Massachusetts, USA.

[bb12] Boisseau, P. & Grodzins, L. (1987). *Hyperfine Interact.* **33**, 283–292.

[bb13] Bonnin, A., Wright, J. P., Tucoulou, R. & Palancher, H. (2014). *Appl. Phys. Lett.* **105**, 084103.

[bb14] Bottelberghs, P. H. & van Buren, F. R. (1975). *J. Solid State Chem.* **13**, 182–191.

[bb15] Cedola, A., Campi, G., Pelliccia, D., Bukreeva, I., Fratini, M., Burghammer, M., Rigon, L., Arfelli, F., Chang Chen, R., Dreossi, D., Sodini, N., Mohammadi, S., Tromba, G., Cancedda, R. & Mastrogiacomo, M. (2014). *Phys. Med. Biol.* **59**, 189–201. 10.1088/0031-9155/59/1/189 24334371

[bb53] Censor, Y., Elfving, T., Herman, G. T. & Nikazad, T. (2007). *SIAM J. Sci. Comput.* **30**, 473–504.

[bb54] Censor, Y., Gordon, D. & Gordon, R. (2001). *Parallel Comput.* **27**, 777–808.

[bb55] Cimmino, G. (1938). *Ric. Sci.* **16**, 326–333.

[bb16] De Nolf, W. & Janssens, K. (2010). *Surf. Interface Anal.* **42**, 411–418.

[bb17] Egan, C. K., Jacques, S. D. M., Di Michiel, M., Cai, B., Zandbergen, M. W., Lee, P. D., Beale, A. M. & Cernik, R. J. (2013). *Acta Biomater.* **9**, 8337–8345. 10.1016/j.actbio.2013.06.018 23791674

[bb18] Elliott, J. C. & Dover, S. D. (1982). *J. Microsc.* **126**, 211–213. 10.1111/j.1365-2818.1982.tb00376.x 7086891

[bb19] Goranson, R. W. & Kracek, F. C. (1935). *J. Chem. Phys.* **3**, 107–115.

[bb20] Gordon, R., Bender, R. & Herman, G. T. (1970). *J. Theor. Biol.* **29**, 471–481. 10.1016/0022-5193(70)90109-8 5492997

[bb21] Grunwaldt, J.-D., Wagner, J. B. & Dunin-Borkowski, R. E. (2013). *ChemCatChem*, **5**, 62–80.

[bb56] Hansen, P. C. & Saxild-Hansen, M. (2012). *J. Comput. Appl. Math.* **236**, 2167–2178.

[bb22] Harding, G., Kosanetzky, J. & Neitzel, U. (1987). *Med. Phys.* **14**, 515–525. 10.1118/1.596063 3626990

[bb23] Haynes, W. M. (2014). *CRC Handbook of Chemistry and Physics*, 95th Ed. Boca Raton: CRC Press.

[bb24] Hiyoshi, N. & Ikeda, T. (2015). *Fuel Process. Technol.* **133**, 29–34.

[bb25] Hou, S., Cao, Y., Xiong, W., Liu, H. & Kou, Y. (2006). *Ind. Eng. Chem. Res.* **45**, 7077–7083.

[bb26] Hounsfield, G. N. (1973). *Br. J. Radiol.* **46**, 1016–1022. 10.1259/0007-1285-46-552-1016 4757352

[bb27] Jacques, S. D., Di Michiel, M., Beale, A. M., Sochi, T., O’Brien, M. G., Espinosa-Alonso, L., Weckhuysen, B. M. & Barnes, P. (2011). *Angew. Chem. Int. Ed.* **50**, 10148–10152. 10.1002/anie.201104604 21936040

[bb60] Jacques, S. D. M., Di Michiel, M., Kimber, S. A. J., Yang, X., Cernik, R. J., Beale, A. M. & Billinge, S. J. L. (2013). *Nat. Commun.* **4**, 2536. 10.1038/ncomms3536 24077398

[bb28] Jensen, K. M. O., Yang, X., Laveda, J. V., Zeier, W. G., See, K. A., Michiel, M. D., Melot, B. C., Corr, S. A. & Billinge, S. J. L. (2015). *J. Electrochem. Soc.* **162**, A1310–A1314.

[bb29] Johansson, G. A., Tyliszczak, T., Mitchell, G. E., Keefe, M. H. & Hitchcock, A. P. (2007). *J. Synchrotron Rad.* **14**, 395–402. 10.1107/S0909049507029962 17717380

[bb30] Kak, A. C. (1979). *Proc. IEEE*, **67**, 1245–1272.

[bb31] Kak, A. C. & Slaney, M. (1988). *Principles of Computerized Tomographic Imaging.* New York: IEEE Press.

[bb57] Landweber, L. (1951). *Am. J. Math.* **73**, 615–624.

[bb32] Lima, C. L., Saraiva, G. D., Freire, P. T. C., Maczka, M., Paraguassu, W., de Sousa, F. F. & Mendes Filho, J. (2011). *J. Raman Spectrosc.* **42**, 799–802.

[bb33] Liu, L. (2014). *J. Med. Imaging Radiat. Sci.* **45**, 131–136. 10.1016/j.jmir.2014.02.002 31051943

[bb34] O’Brien, M. G., Jacques, S. D. M., Di Michiel, M., Barnes, P., Weckhuysen, B. M. & Beale, A. M. (2012). *Chem. Sci.* **3**, 509–523.

[bb35] Palancher, H., Tucoulou, R., Bleuet, P., Bonnin, A., Welcomme, E. & Cloetens, P. (2011). *J. Appl. Cryst.* **44**, 1111–1119.

[bb58] Pan, S. X. & Kak, A. C. (1983). *IEEE Trans. Acoust. Speech Signal Process.* **31**, 1262–1275.

[bb36] Pistorius, C. W. F. T. (1966). *J. Chem. Phys.* **44**, 4532–4537.

[bb37] Price, S. W. T., Geraki, K., Ignatyev, K., Witte, P. T., Beale, A. M. & Mosselmans, J. F. W. (2015). *Angew. Chem.* **127**, 10024–10027. 10.1002/anie.201504227 PMC4600245 26140613

[bb38] Ruiz-Martínez, J., Beale, A. M., Deka, U., O’Brien, M. G., Quinn, P. D., Mosselmans, J. F. W. & Weckhuysen, B. M. (2013). *Angew. Chem. Int. Ed.* **52**, 5983–5987. 10.1002/anie.201210030 PMC3749464 23616490

[bb39] Sadjadi, S., Jašo, S., Godini, H. R., Arndt, S., Wollgarten, M., Blume, R., Görke, O., Schomäcker, R., Wozny, G. & Simon, U. (2015). *Catal. Sci. Technol.* **5**, 942–952.

[bb40] Schroer, C. G., Kuhlmann, M., Günzler, T. F., Lengeler, B., Richwin, M., Griesebock, B., Lützenkirchen-Hecht, D., Frahm, R., Ziegler, E., Mashayekhi, A., Haeffner, D. R., Grunwaldt, J.-D. & Baiker, A. (2003). *Appl. Phys. Lett.* **82**, 3360–3362.

[bb41] Stock, S. R. & Almer, J. D. (2012). *J. Appl. Cryst.* **45**, 1077–1083.

[bb42] Stock, S. R., De Carlo, F. & Almer, J. D. (2008). *J. Struct. Biol.* **161**, 144–150. 10.1016/j.jsb.2007.10.001 18006333

[bb43] Toby, B. H. & Von Dreele, R. B. (2013). *J. Appl. Cryst.* **46**, 544–549.

[bb44] Valentini, L., Artioli, G., Voltolini, M. & Dalconi, M. C. (2012). *J. Am. Ceram. Soc.* **95**, 2647–2652.

[bb45] Valentini, L., Dalconi, M. C., Parisatto, M., Cruciani, G. & Artioli, G. (2011). *J. Appl. Cryst.* **44**, 272–280.

[bb46] Vamvakeros, A., Jacques, S. D. M., Di Michiel, M., Middelkoop, V., Egan, C. K., Cernik, R. J. & Beale, A. M. (2015). *J. Appl. Cryst.* **48**, 1943–1955. 10.1107/S160057671600131X PMC4815873 27047305

[bb47] Vamvakeros, A., Jacques, S. D. M., Middelkoop, V., Di Michiel, M., Egan, C. K., Ismagilov, I. Z., Vaughan, G. B. M., Gallucci, F., van Sint Annaland, M., Shearing, P. R., Cernik, R. J. & Beale, A. M. (2015). *Chem. Commun.* **51**, 12752–12755. 10.1039/c5cc03208c 26041252

[bb48] Vanmeert, F., Van der Snickt, G. & Janssens, K. (2015). *Angew. Chem. Int. Ed.* **54**, 3607–3610. 10.1002/anie.201411691 25703204

[bb49] Voltolini, M., Dalconi, M. C., Artioli, G., Parisatto, M., Valentini, L., Russo, V., Bonnin, A. & Tucoulou, R. (2013). *J. Appl. Cryst.* **46**, 142–152.

[bb50] Wang, Y., Jacobsen, C., Maser, J. & Osanna, A. (2000). *J. Microsc.* **197**, 80–93. 10.1046/j.1365-2818.2000.00629.x 10620151

[bb51] Wragg, D. S., O’Brien, M. G., Di Michiel, M. & Lønstad-Bleken, F. (2015). *J. Appl. Cryst.* **48**, 1719–1728.

