# Supplementary material for: Interlaced X-ray diffraction computed tomography
Source: J Appl Crystallogr. 2016 Mar 1;49(Pt 2):485–96. doi: 10.1107/S160057671600131X (PMC4815873; doi:10.1107/S160057671600131X)
Supplement: Supplementary file 1 [file j-49-00485-sup1.pdf]

Interlaced X-ray diffraction computed tomography

Authors

Antonios Vamvakeros[ab], Simon D. M. Jacques[abc]*, Marco Di Michiel[d], Pierre Senecal[ab], Vesna Middelkoop[e], Robert J. Cernik[c] and Andrew M. Beale[ab]*

[a]Department of Chemistry, University College London, 20 Gordon Street, London, WC1H 0AJ, UK

[b]Research Complex at Harwell, Rutherford Appleton Laboratory, Didcot, Harwell, Oxfordshire, OX11 0FA, UK

[c]School of Materials, University of Manchester, Manchester, Lancashire, M13 9PL, UK

[d]ESRF - The European Synchrotron, Grenoble, F-38000, France

[e]Flemish Institute for Tecnological Research, VITO NV, Boeretang 200, Mol, Belgium

Correspondence email: simon.jacques@gmail.com; andrew.beale@ucl.ac.uk

1. Sampling in the frequency domain

As discussed in the main paper, the artefacts shown in Figure 5 are a result of undersampling during the tomographic scan. An illustration of this problem for traditional X-ray CT using a parallel X-ray beam is provided in Figure S1. If the number of projections is small, then the θ angle in the Fourier space will be large, which will lead to aliasing distortions in the reconstructed images. This phenomenon is thoroughly discussed in the work of Pan and Kak (Pan & Kak, 1983).

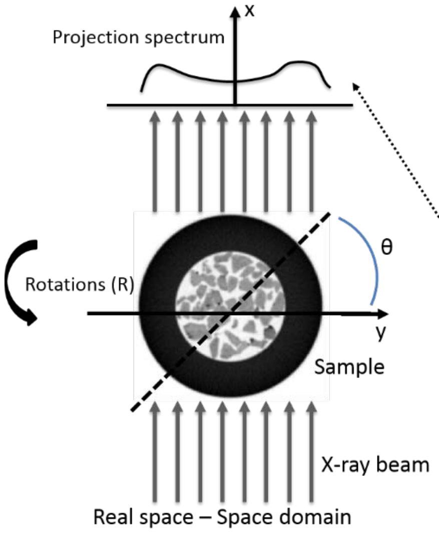

Figure S1 The sampling in the real space and frequency domain during a tomographic scan.

2. Comparison of different reconstruction algorithms

In Figure S2, the reconstructed images of the sinograms presented in Figure 5 of the main paper are shown. For comparison, both the filtered back projection (FBP) and the SART algorithm have been implemented to reconstruct the images (Kak, 1979, Andersen & Kak, 1984). The built-in function *iradon* of MATLAB (The MathWorks Inc., Natick, MA, USA) was used for the FBP algorithm and the AIR Tools package (a MATLAB package with implementations of several algebraic iterative reconstruction methods) was used for the SART algorithm (Hansen & Saxild-Hansen, 2012). The FBP reconstructed image was used for the first iteration (instead of a zeros matrix) in order to reduce the computational time for the SART algorithm (i.e. achieving the same final reconstructed images with less iterations). In Figure S2, it can be clearly seen that the quality of the reconstructed images corresponding to spatial resolution *2x*, *4x* and *8x* does not improve further when the SART algorithm is used regardless how many iterations are applied. In the case of the spatial resolution *1x* sinogram (angular step size of 12 °), the SART algorithm seems to supress the aliasing artefacts but at expense of spatial information. For example, small catalyst particles disappear with increasing number of iterations (as indicated by the arrows in Figure S2).

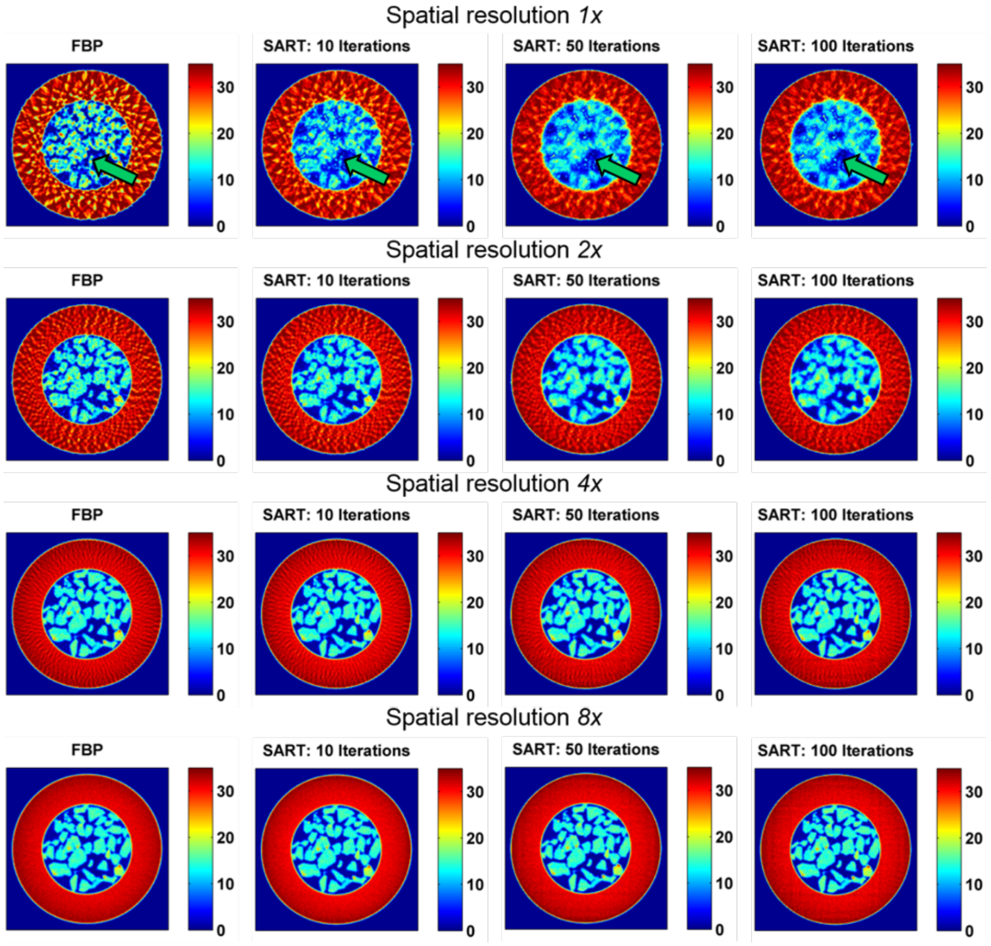

Figure S2 Reconstructed images of the sinograms presented in Figure 5 of the main paper using the filtered back projection (FBP) and the SART algorithms (10, 50 and 100 iterations).

A number of algebraic iterative reconstruction techniques were tested for the spatial resolution *1x* sinogram and the results are presented in Figure S3. More specifically, the SART algorithm, the diagonally relaxed orthogonal projections (DROP) method, Landweber's method, Cimmino's method and the Component Averaging (CAV) method were tested as provided by the AIR Tools package (Andersen & Kak, 1984, Censor *et al.*, 2007, Landweber, 1951, Cimmino, 1938, Censor *et al.*, 2001). It can be seen that these reconstruction algorithms yield almost identical images. In this study it was chosen to convolute the sinograms with an appropriate window function and use the FBP for reconstruction as discussed in the main paper.

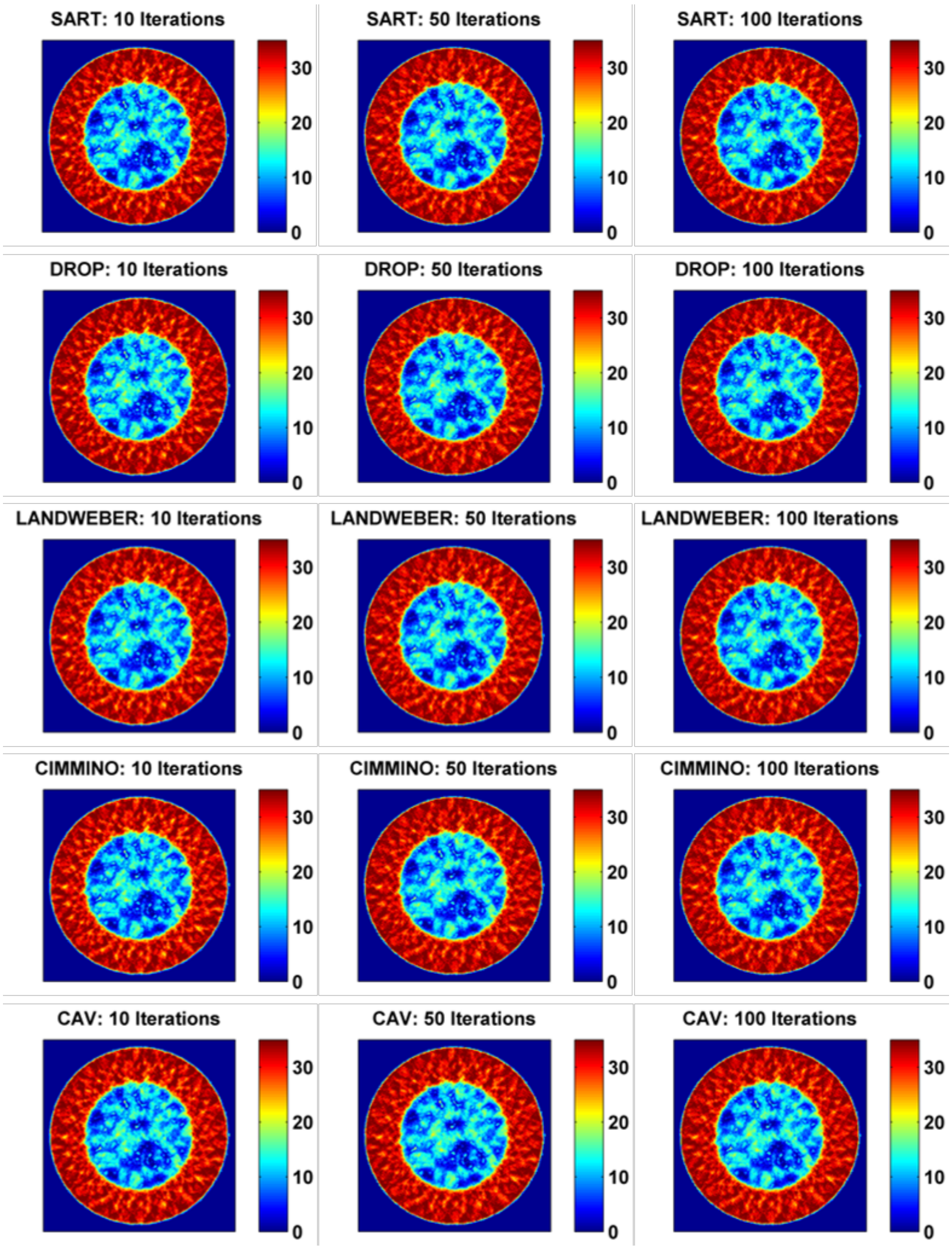

Figure S3 Reconstructed images of the sinogram corresponding to spatial resolution *1x* presented in Figure 5 of the main paper using the SART, DROP, Lanweber, Cimmino and CAV algorithms (10, 50 and 100 iterations).

3. Catalyst preparation

The 2%Mn-1.6%Na-3.1%W/SiO_2 catalyst was prepared by a sequential incipient wetness impregnation method. Firstly, the SiO_2 support (Silica gel Davisil 646, ~ 250-500 μm) was impregnated by an aqueous solution of sodium tungstate dihydrate $Na_2WO_4 \cdot 2H_2O$ and sodium oxalate $Na_2C_2O_4$ salts taken in appropriate concentrations at a temperature of 80ºC. The Na-W/SiO_2 was dried at 120 ºC for 6 h and was then impregnated by an aqueous solution of manganese (II) acetate tetrahydrate $Mn(CH3COO)_2 \cdot 4H_2O$ salt.

4. Solid state changes as a function of temperature

The summed diffraction patterns for every translational (line) scan for the two IXRD-CT scans are plotted in Figure S4. In total, 240 diffraction patterns have been plotted (i.e. 120 per IXRD-CT scan). As expected, the signal from the capillary contributes to the formation of a high intensity background. An appropriate polynomial was fitted with MATLAB and the background was subtracted as shown in Figure S4.

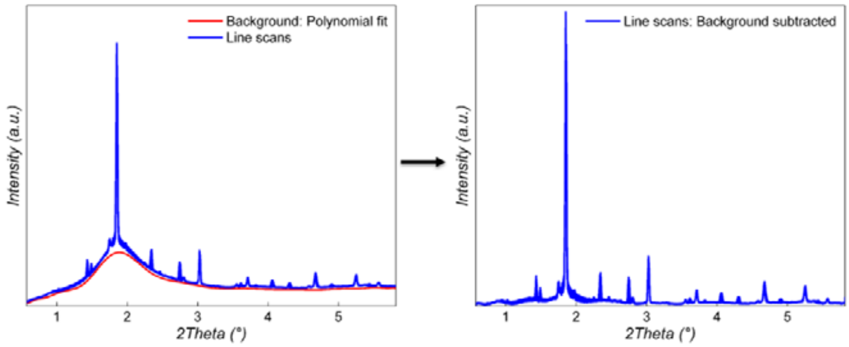

Figure S4 Left: The summed diffraction patterns for every translational scan for the two IXRD-CT scan (240 in total) and the polynomial created for background subtraction are presented. Right: The 240 diffraction patterns after the background subtraction are shown.

The 240 diffraction patterns after the background subtraction are plotted as a function of temperature in Figure S5. It can be seen that the peaks corresponding to cubic (Fd-3m) Na_2WO_4 (e.g. 1.42 ° 2θ) disappear at approximately 600 °C.

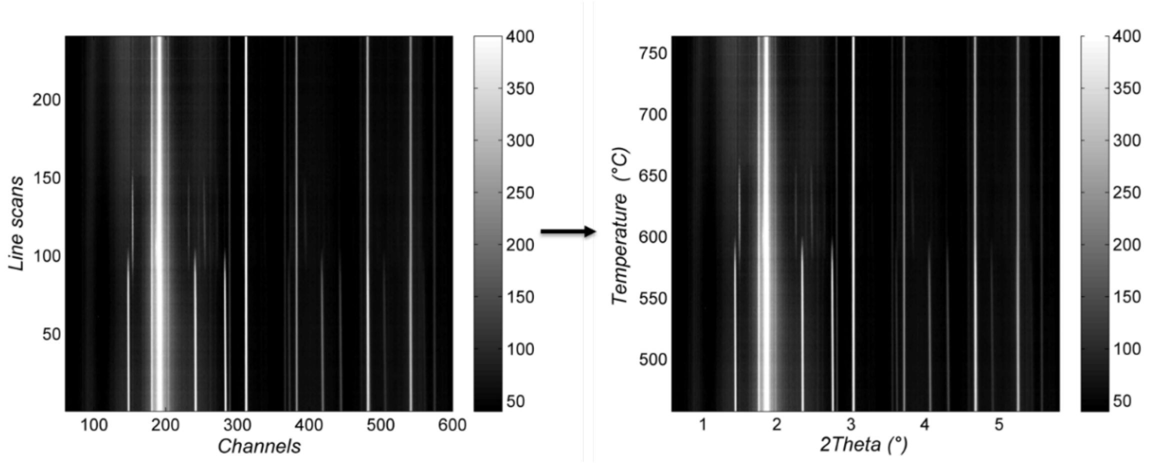

Figure S5 Left: The 240 diffraction patterns after the background subtraction are plotted. Right: These diffraction patterns are plotted as a function of temperature.

5. Pawley whole powder pattern fitting

As mentioned in the main paper, the main crystalline phases present in the catalyst, in the temperature range of 620 to 640 °C, are cristobalite, tridymite, Na_2WO_4, and Mn_2O_3. There are also minor peaks of quartz and $Na_2Si_2O_5$ (as a result of the excess of Na during the catalyst preparation). As recently reported by Sadjadi et al., it is not trivial to decide whether the Mn_2O_3 and/or the Mn_7SiO_{12} phases are present in the catalyst as both generate similar diffraction patterns (Sadjadi *et al.*, 2015). As a result, all the previously mentioned phases were used for the whole powder pattern fitting. Pawley analysis was performed with the GSASII software using the appropriate unit cells: tetragonal I-42d for cristobalite (ICSD ref: 44095), hexagonal $P6_3/mmc$ for tridymite (ICSD ref: 200479), cubic Ia-3 for Mn_2O_3 (ICSD ref: 187263), $P6_222$ for quartz (ICSD ref: 89287), $I4_1/acd$ for Mn_7SiO_{12} (ICSD ref: 12123), monoclinic $P2_1/n$ for $Na_2Si_2O_5$ (ICSD ref: 88662) and orthorhombic Fddd for the low symmetry Na_2WO_4 phase. The summed diffraction from tomo scan 1 of IXRD-CT scan 2 was used for the Pawley analysis, corresponding to a temperature range of 620 to 640 °C, as there is only the low symmetry Na_2WO_4 phase present and no cubic Na_2WO_4. The results are presented in Figure S6 where it can be seen that all the peaks present in the diffractogram have been fitted.

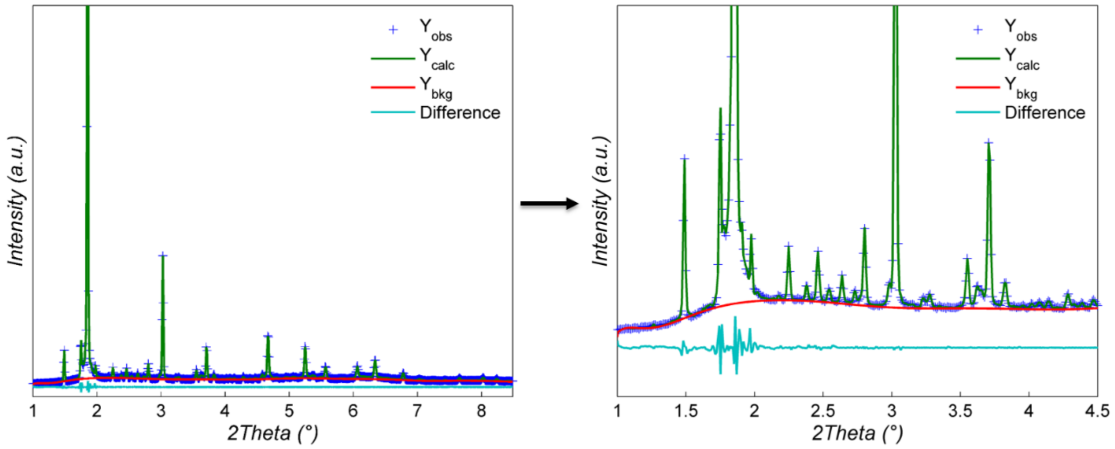

Figure S6 Left: Pawley whole powder pattern profile fitting using the summed diffraction pattern of tomo scan 1 of IXRD-CT scan 2. Right: A region of interest showing that even the minor peaks present in the diffractogram have been fitted appropriately.

References

Andersen, A. H. & Kak, A. C. (1984). *Ultrasonic Imaging* **6**, 81-94.
Censor, Y., Elfving, T., Herman, G. T. & Nikazad, T. (2007). *SIAM Journal on Scientific Computing* **30**, 473-504.
Censor, Y., Gordon, D. & Gordon, R. (2001). *Parallel Computing* **27**, 777-808.
Cimmino, G. (1938). *La Ricerca Scientifica* **16**, 326-333.
Hansen, P. C. & Saxild-Hansen, M. (2012). *Journal of Computational and Applied Mathematics* **236**, 2167-2178.
Kak, A. C. (1979). *Proceedings of the IEEE* **67**, 1245-1272.
Landweber, L. (1951). *American Journal of Mathematics* **73**, 615-624.
Pan, S. X. & Kak, A. C. (1983). *Acoustics, Speech and Signal Processing, IEEE Transactions on* **31**, 1262-1275.
Sadjadi, S., Jašo, S., Godini, H. R., Arndt, S., Wollgarten, M., Blume, R., Görke, O., Schomäcker, R., Wozny, G. & Simon, U. (2015). *Catalysis Science and Technology* **5**, 942-952.
